# Visual and label-free ASFV and PCV2 detection by CRISPR-Cas12a combined with G-quadruplex

**DOI:** 10.3389/fvets.2022.1036744

**Published:** 2022-11-29

**Authors:** Ying Wang, Rong Li, Yang Zhang, Weida Zhang, Sishun Hu, Zili Li

**Affiliations:** ^1^State Key Laboratory of Agricultural Microbiology, College of Veterinary Medicine, Huazhong Agricultural University, Wuhan, China; ^2^College of Veterinary Medicine, Shanxi Agricultural University, Jinzhong, China; ^3^Key Laboratory of Preventive Veterinary Medicine in Hubei Province, Wuhan, China; ^4^Key Laboratory of Development of Veterinary Diagnostic Products, Ministry of Agriculture of the People's Republic of China, Wuhan, China

**Keywords:** ASFV, PCV2, detection, Cas12a, G-quadruplex

## Abstract

African swine fever (ASF) and postweaning multisystemic wasting syndrome (PMWS) are acute infectious diseases caused by the African swine fever virus (ASFV) and porcine circovirus type 2 (PCV2). At present, there are no effective vaccines for the prevention of ASFV. PMWS, which is harmful to the domestic and even the world pig industry, is difficult to cure and has a high mortality. So, developing simple, inexpensive, and accurate analytical methods to detect and effectively diagnose ASFV and PCV2 can be conducive to avoid ASFV and PCV2 infection. CRISPR has become a potentially rapid diagnostic tool due to recent discoveries of the trans-cleavage properties of CRISPR type V effectors. Herein, we report the visual detection based on CRISPR-Cas12a (cpf1), which is more convenient than fluorescence detection. Through *in vitro* cleavage target DNA activation, Cas12a can trans-cleavage ssDNA G-quadruplex. TMB/H_2_O_2_ and Hemin cannot be catalyzed by cleavaged G-DNA to produce green color products. This protocol is useful for the detection of ASFV and PCV2 with high sensitivity. This method can enable the development of visual and label-free ASFV and PCV2 detection and can be carried out in the field without relying on instruments or power. This method can complete nucleic acid detection at 37 °C without using other instruments or energy. Our research has expanded the application of Cas12a and laid the foundation for the field's rapid detection of viral nucleic acid in future.

## Introduction

African swine fever (ASF) and porcine circovirus-associated diseases are infectious diseases caused by the African swine fever virus (ASFV) and porcine circovirus. ASFV's clinical symptoms vary from acute and subacute to chronic. It is characterized by high fever, cyanosis, widespread internal organ bleeding, respiratory disorder, and neurological symptoms. It is incidence and mortality rate are nearly 100% (1). ASF has broken out in Europe, Africa, and Asia and brought huge economic losses to the pig industry (2, 3). ASF was first reported in China in 2018 and has caused huge social and economic losses (4, 5). Recently, the low pathogenicity of ASFV non-hemadsorbing isolates has been reported (6), which makes the prevention and control of ASF more and more difficult. Real-time polymerase chain reaction (PCR) assay and chemiluminescence immunoassay have been reported for the detection of ASFV (7, 8).

Porcine circovirus type 2 (PCV2) is classified in the circoviridae family and ssDNA animal virus (9) and is associated with postweaning multisystemic wasting syndrome (PMWS) in the pig. PCV2 has caused severe losses in the global swine industry in recent decades (10). Although the PCV2 vaccine is available because of the short protection period and mixed infection of different genotypes, it is not easy to eliminate PCV2 from pigs by vaccination alone (11, 12). Loop-mediated isothermal amplification (LAMP) and multiplex real-time PCR assay have been used for PCV2 detection (13, 14).

Developing simple, inexpensive, and accurate analytical methods to detect and effectively diagnose ASFV and PCV2 is conducive to avoid ASFV and PCV2 infection. The diagnosis of virus infection generally includes virus isolation and identification, the detection of virus nucleic acid, antigen, and the specific antibody, which not only takes a long time but also requires high standards of medical equipment and operators. Ideal diagnostic tests should deliver results quickly and be enabled for instant use on a variety of sample types without excessive reliance on a technician or an auxiliary device (15).

At the University of California, Jennifer Doudna, Alexandra East-Seletsky, and their colleagues used Cas13a incidental cleavage activity to detect RNA (16). Zhang Feng has demonstrated that Cas13-based Specific High Sensitivity Enzymatic Reporter UnLOCKing (SHERLOCK) can be applied for detecting Zika virus (ZIKV) and Dengue virus (DENV) in patient's samples (17). When Cas13a detects the target RNA sequence, its indistinguishable RNAase activity (incidental cleavage activity) also cleaves the RNA reporter molecule and releases detectable fluorescent signals.

When Cas13a binds to crRNA and identifies the corresponding RNA sequence, Cas13a RNAase activity is activated, and the activity of Cas13a also cuts other untargeted RNA. This phenomenon is called the “collateral effect.” At the same time, Chinese and American researchers proposed that FnCpf1 and LbCpf1 proteins (also known as FnCas12a and LbCas12a) also have the “collateral effect” (18). These gene detection techniques based on LbCas12a are named DETECTR (DNA Endonuclease Targeted CRISPR Trans Reporter). The principle of DETECTR is similar to SHERLOCK. Cas13a recognizes target RNA and is activated for untargeted RNA cleavage, while Cas12a recognizes target DNA and is activated for untargeted ssDNA cleavage. F-Q label RNA/ssDNA cleavage by Cas13a/12a is confirmed by fluorescence detection.

DNAzymes possess practical advantages (19), DNA hybridization assays, and catalytic beacons for the detection of DNA and telomerase activity (20). As for metal ion detection, K(+) sensitive G-quadruplex DNA PS5.M as a sensitive element has promoted the development of K(+) detection (21). Aptasensors for small molecules and proteins, utilizing the bioelectrocatalytic function of hemin/G-quadruplex DNase activity to develop glucose oxidation and biosensors detection methods also can be used to detect DNA or low-molecular-weight substrates (22). For example, visual detection of single-nucleotide polymorphisms (SNP) (23). Therefore, they can be promising candidates for practical applications.

Herein, we report the visual detection based on CRISPR-Cas12a (cpf1), which is more convenient than fluorescence detection. Through *in vitro* cleavage target DNA activation Cas12a trans-cleavage ssDNA G-quadruplex. TMB/H_2_O_2_ and Hemin cannot be catalyzed by cleavage G-DNA to produce green color products. This protocol is useful for the detection of ASFV and PCV2 nucleic acid with high sensitivity. As demonstrated, our research could enable the development of visual and label-free ASFV detection and can be carried out in the field without relying on instruments or power.

## Materials and methods

The short G-rich DNA sequence (GDS) PW17 5' GGGTAGGGCGGGTTGGG 3' and primers used in this study were synthesized and provided by Tsingke Biotech Co. (Beijing, China) (Supplementary Table S1). Hemin, Tris–HCl, DMSO (Dimethyl Sulfoxide), and KCl were purchased from Sigma-Aldrich (Shanghai, China). TwistAmp^®^ Basic recombinase polymerase amplification (RPA) was used to amplify clinical samples. LbCas12a was purchased from NEB (M0653T, Beijing, NEB). T7 high-yield RNA transcription kit was purchased from Vazyme (Nanjing, China). 10 × reaction buffer prepared with 100 mM Tris–HCl, 150 mM NaCl, 12 mM MgCl_2_, and 12 mM KCl.

### Determination of the optimum hemin and G-quadruplex concentration

PW17 is a nucleic acid sequence often used to form G-quadruplex monomer. Synthetic PW17 was dissolved in 100 μM DNA stock solution by ddH_2_O. PW17 was diluted before use. Hemin was dissolved with DMSO to 50 mM, stored at 4 °C, and diluted before use. In order to determine the optimum hemin concentration, 1.0 μM G-quadruplex and different concentrations of hemin were mixed, respectively, with reaction buffer at a total volume of 50 μL at 37 °C for 60 min. For checking the optimum G-quadruplex concentration, 2.0 μM hemin was mixed with different G-quadruplex concentrations, respectively. The reaction system is the same as above. The optimal reaction time was measured to determine the best detection time. We mixed 2.0 μM hemin with 0.4 μM G-quadruplex at 37 °C, observed the color, and determined the OD_450_ at different times.

### ASFV and PCV2 target DNA selected and crRNA designed

The African swine fever virus VP72 protein gene and PCV2 capsid protein (Cap protein) gene are the conserved gene sequences selected as target DNAs. The target DNAs were cloned by PCR with primer pairs ASFV VP72 F/R and PCV2 Cap F/R (Supplementary Table S1).

CRISPR-DT online design website was used for crRNA prediction (24). crRNA F and PCV2 crRNA R primers were used for synthesizing crRNA, which targets PCV2. crRNA F and ASFV crRNA R primers were used for synthesizing crRNA, which targets ASFV. The T7 high-yield RNA transcription kit for RNA synthesis *in vitro*.

### Analysis of crRNA cleavage efficiency and optimal cleavage time

LbCas12a and crRNA cleaving target DNA activity *in vitro* were verified by the following reaction system: 500 ng DNA fragment, reaction buffer (10×) 2.5 μL, crRNA 1 μL (40 ng/μL final), 1 μM LbCas12a (Cpf1) 2.5 μL (100 nM final), total reaction volume 25 μL at 37°C for 60 min, and 72°C for 10 min inactivation. Gel electrophoresis is used for the analysis of cleaving efficiency.

A concentration of 50 nM ASFV VP72 DNA fragment, 2.5 μL of cleavage buffer (10×), 1 μL of crRNA at a final concentration of 40 ng/μL, 2.5 μL of 1 μM LbCas12a (Cpf1) at a final concentration of 100 nM, and 1 μL of G-quadruplex and added up to 25 μL by H_2_O at 37 °C for different time periods. Analysis of cutting efficiency is determined by adding 2 μM of hemin, reaction buffer, and 100 μL of TMB/H_2_O_2_. The reaction solution was incubated at 37 °C for 20 min. The color and OD_450_ were observed and determined at different times.

### RPA amplification and nucleic acid extraction

Recombinase polymerase amplification was used to amplify clinical samples *in vitro* (25). TwistAmp^®^ Basic was applied for amplification. PCV2 RPA F/R and ASFV RPA F/R primer pairs were used for RPA. PCV2 Cap and ASFV VP72 DNA templates were adjusted to 1.0 × 10^3^, 1.0 × 10^2^, and 1.0 × 10^1^ copies, and 2 μL of DNA template was added for RPA amplification. MagBead DNA Purification Kit was applied for the purification of the DNA fragments, which were resuspended and recovered with 20 μL of ddH_2_O.

### Sensitivity of PCV2 and ASFV DNA detection by CRISPR-Cas12a combined with G-quadruplex

Porcine circovirus type 2 capsid and ASFV VP72 DNA templates were adjusted to 1.0 × 10^3^, 1.0 × 10^2^, and 1.0 × 10^1^ copies, and 2 μL of DNA template was added for RPA amplification. DNA fragments were purified by resuspending and recovering by 20 μL H_2_O. The 18 μL of RPA reaction product was added with 2.5 μL of reaction buffer (10×), 1 μL of crRNA at a concentration of 40 ng/μL, 2.5 μL of LbCas12a (Cpf1) at a concentration of 100 nM, and 1 μL of G-quadruplex and incubated at 37 °C for 45 min. 2.0 μM hemin, reaction buffer, and 100 μL TMB were added and incubated for 20 min. The observation of green-colored oxidized products determined DNA detection.

### Gel electrophoresis

Gel electrophoresis was to confirm the PCR and RPA amplification and DNA cleavage. The 40 mL agarose gel contains 1.2% agarose and 1×TBE at pH 8.0. The electrophoresis was run with a voltage of 120 V for 30 min.

## Results

### Determination of the optimum hemin and G-quadruplex concentration

**Figure 1:**
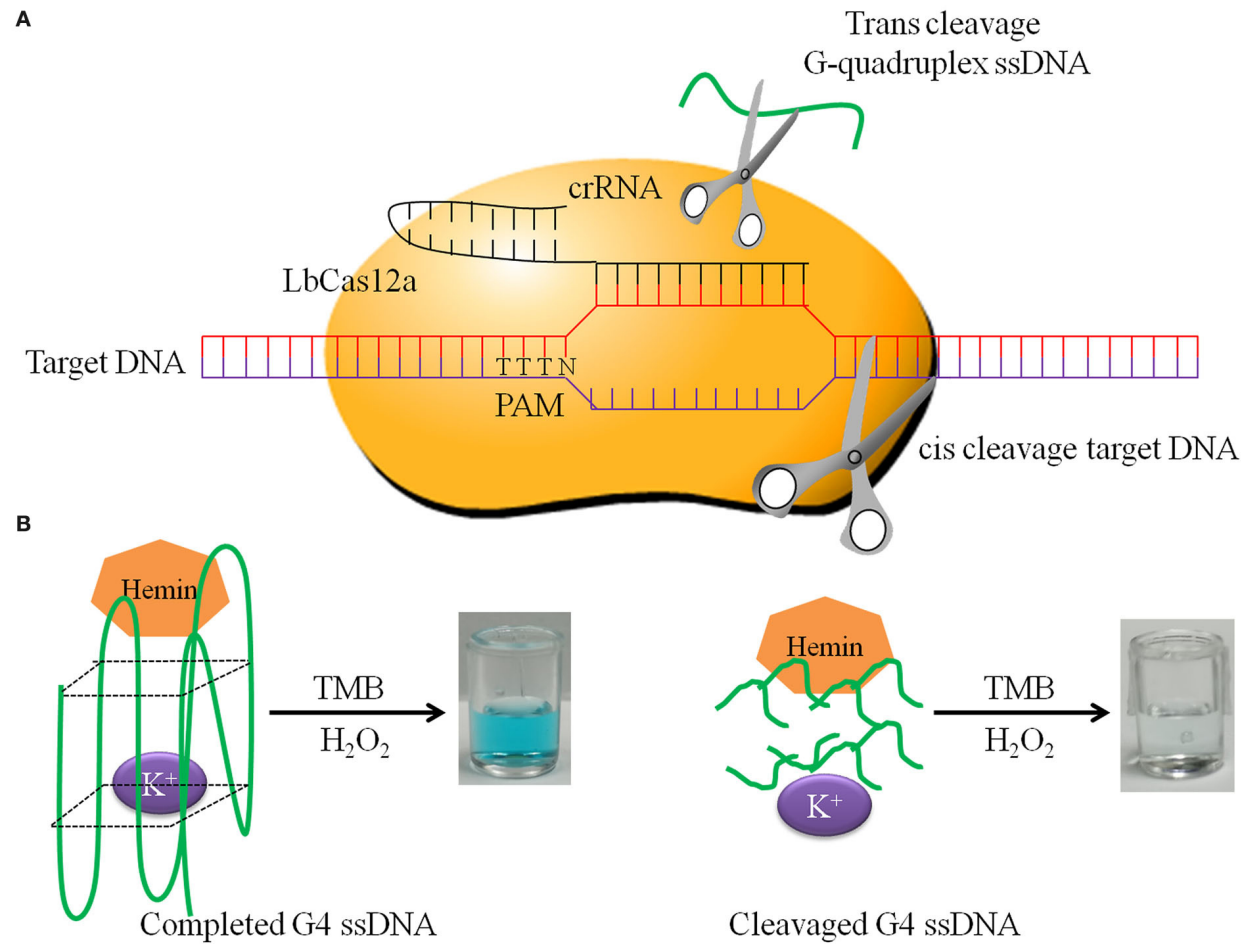
The principle of DNA detection by CRISPR-Cas12a. **(A)** LbCas12a cis-cleavage target DNA and trans-cleavage non-specific G-quadruplex ssDNA. **(B)** Completed G-quadruplex ssDNA has HRP activity in the presence of K^+^ and hemin, which has the ability to catalyze TMB and H_2_O_2_ to form green products. In contrast, cleavaged G-quadruplex ssDNA does not have this ability.

**Figure 2:**
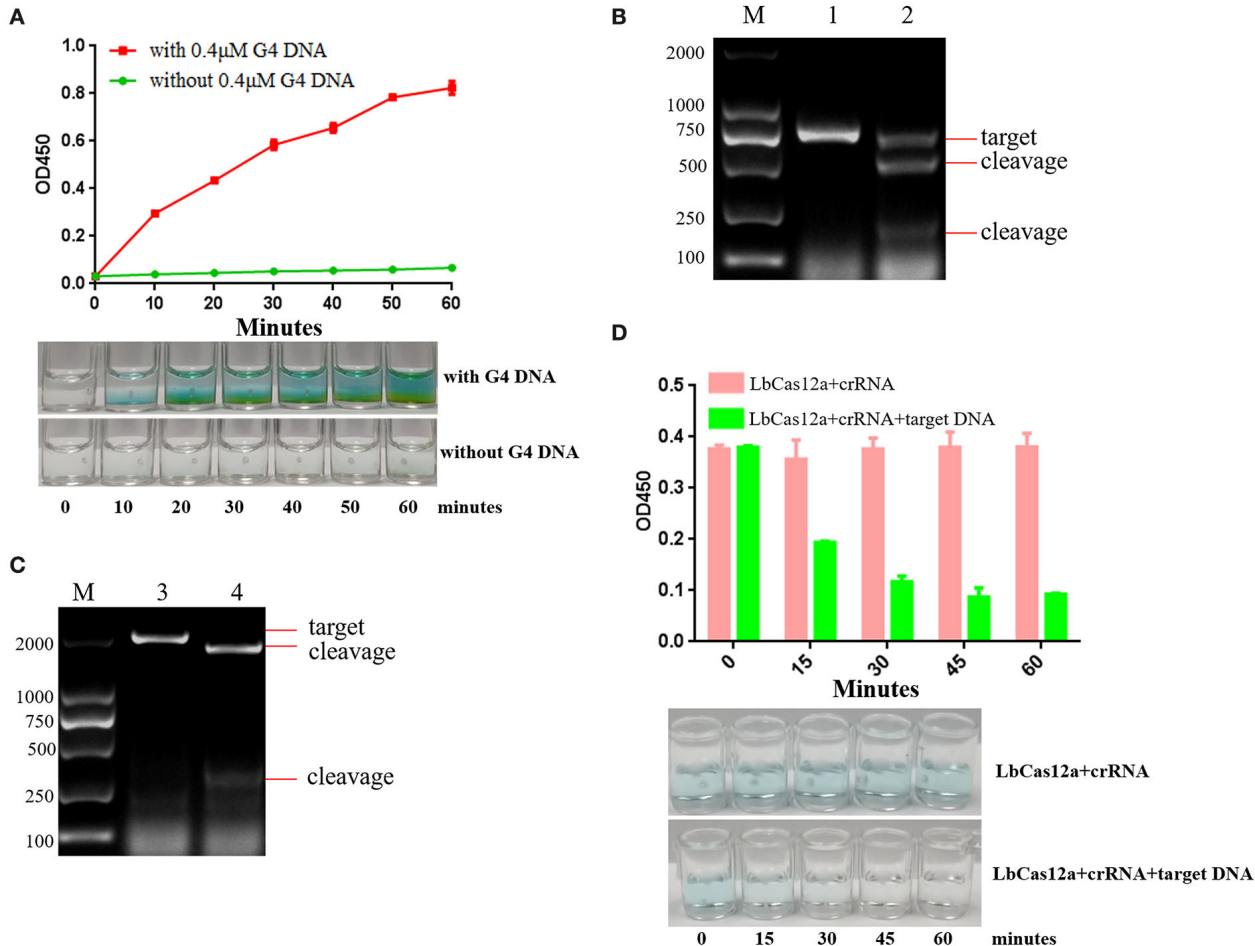
The analysis of optimal reaction time and target DNA sequence. **(A)** TMB is catalyzed by 0.4 μM G-quadruplex and 2 μM hemin. The color gradually deepened with the increase of time, and the green color could be observed after incubation at 37 °C for 20 min. **(B)** crRNAs were designed and synthesized according to the PCV2 Cap protein gene, and the cleavage efficiency of crRNA targeting PCV2 Cap was analyzed. Lane M: 2000 DNA ladder; Lane 1: negative control, PCV2 Cap gene was cut by ASFV crRNA and Lbcas12a; Lane 2: PCV2 Cap gene was cut by PCV2 crRNA and Lbcas12a. **(C)** crRNA was designed and synthesized according to ASFV VP72 protein gene, and the cleavage efficiency of crRNA targeting ASFV VP72 was analyzed. Lane M: 2000 DNA ladder; Lane 3: negative control, ASFV VP72 gene was cut by PCV2 crRNA and Lbcas12a; Lane 4: ASFV VP72 gene was cut by ASFV crRNA and Lbcas12a. **(D)** Evaluation of the efficiency of trans-cleavage G-quadruplex ssDNA by LbCas12a with crRNA over time.

Cas12a, upon cleaving the target dsDNA, will proceed to cleave ssDNA in a nonspecific manner, the so-called “trans-cleavage.” Using this principle, the target sequence, amplificated by RPA, is cut by Cas12a and crRNA, activating the trans-cleavage activities of Cas12a. The G-quadruplex ssDNA is cut by activated Cas12a, so it cannot form a spatial structure and loses the oxidase activity. If there is no target sequence, the G-quadruplex ssDNA is not cut by Cas12a. Then G-quadruplex forms a spatial structure and has oxidase activity. The complete G-quadruplex and hemin combine to catalyze TMB to show blue. In this study, the positive reaction is colorless, and the negative reaction is blue. The principle of DNA detection by CRISPR-Cas12a and the research scheme of this study is shown in Figure 1. When the OD_450_ was close to 1.0, the minimum hemin concentration was the best dosage. We determined that the optimal hemin concentration was 2.0 μM (Supplementary Figure S1). When the reaction was obviously green, the minimum G-quadruplex concentration was selected as the best dosage. The optimal G-quadruplex concentration was 0.4 μM (Supplementary Figure S2). With the prolongation of reaction time, the color green gradually deepened, and OD increased; the figure shows the color development over the time period of 60 min (Figure 2A).

### ASFV VP72 protein gene and PCV2 cap protein gene as the target DNAs

VP72 and Cap are the conserved genes of ASFV and PCV2. To investigate the DNA cleavage feature of Cas12a, we employed LbCas12a to cleave target DNA *in vitro*. ASFV VP72 protein gene and PCV2 Cap protein gene are the target DNAs and were cloned (Supplementary Figure S3) by PCR with primer pairs PCV2 Cap F/R and ASFV VP72 F/R.

### crRNA cutting efficiency and optimal cleavage time

CRISPR-DT online design website was used for crRNA prediction. Two crRNAs were designed (Supplementary Figures S4, S5) and synthesized, respectively, in each target DNA with primer crRNA F and PCV2 crRNA R/ASFV crRNA R by T7 high-yield RNA transcription kit. The activity of LbCas12a and crRNA cleaving target DNA *in vitro* was verified. The results are presented in Figures 2B,C. In the presence of LbCas12a and crRNA, the PCV2 Cap gene and ASFV VP72 gene target sequences were cut and generated short products. The results indicated that target DNAs were recognized by designing crRNA efficiently.

Single-stranded DNA can be degraded by activated Cas12a non-specifically. LbCas12a and crRNA cutting PW17 G-quadruplex ssDNA activity *in vitro* was verified. The results indicated that G-quadruplex DNA was cut by activated LbCas12a. Once generated cleavage G-quadruplex, the green color will not be observed. The best cleavage efficiency can be achieved in 45 min (Figure 2D). Over time, the green color could not be observed. In order to ensure the completion of G-quadruplex cleavage, 45 min had been selected as the reaction cleavage time.

### Sensitivity of PCV2 and ASFV DNA detection by CRISPR-Cas12a combined with G-quadruplex

LbCas12a is a fluorescence analyzer for gene detection, so it is important to develop a detection method for LbCas12a nucleic acid without power or instruments. Activated Cas12a can cut G-quadruplex and make the color reaction disappear. It can be used for unmarked naked-eye detection.

**Figure 3:**
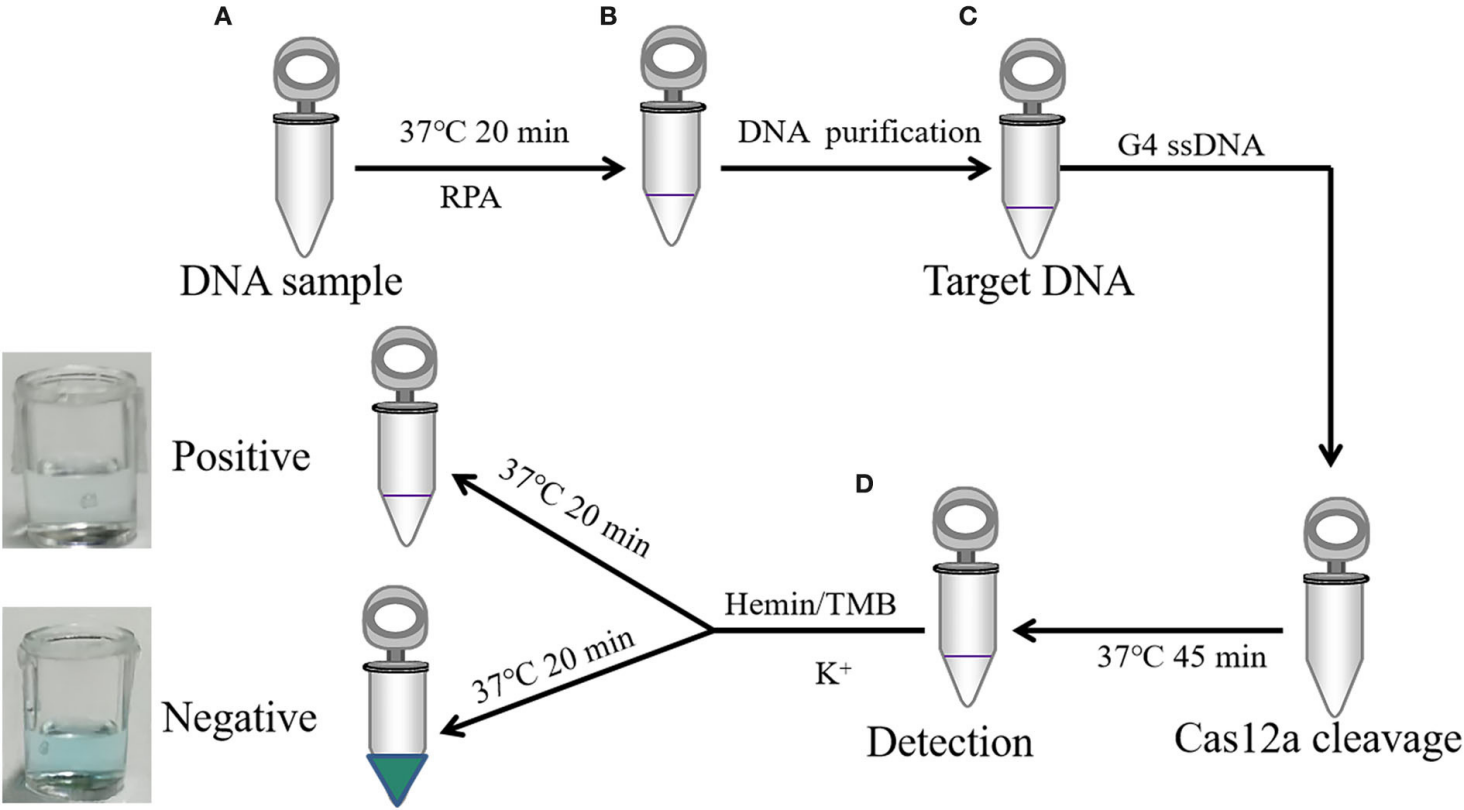
A schematic illustration of DNA detection by CRISPR-Cas12a combined with G-quadruplex. **(A)** RPA amplified target sequence. **(B)** Target DNA magnetic bead purification. **(C)** LbCas12a cleavage target DNA and non-specific cleavage G-quadruplex ssDNA incubated at 37 °C for 60 min. **(D)** Color observation after incubation adding hemin, TMB, and K^+^ at 37 °C for 20 min.

**Figure 4:**
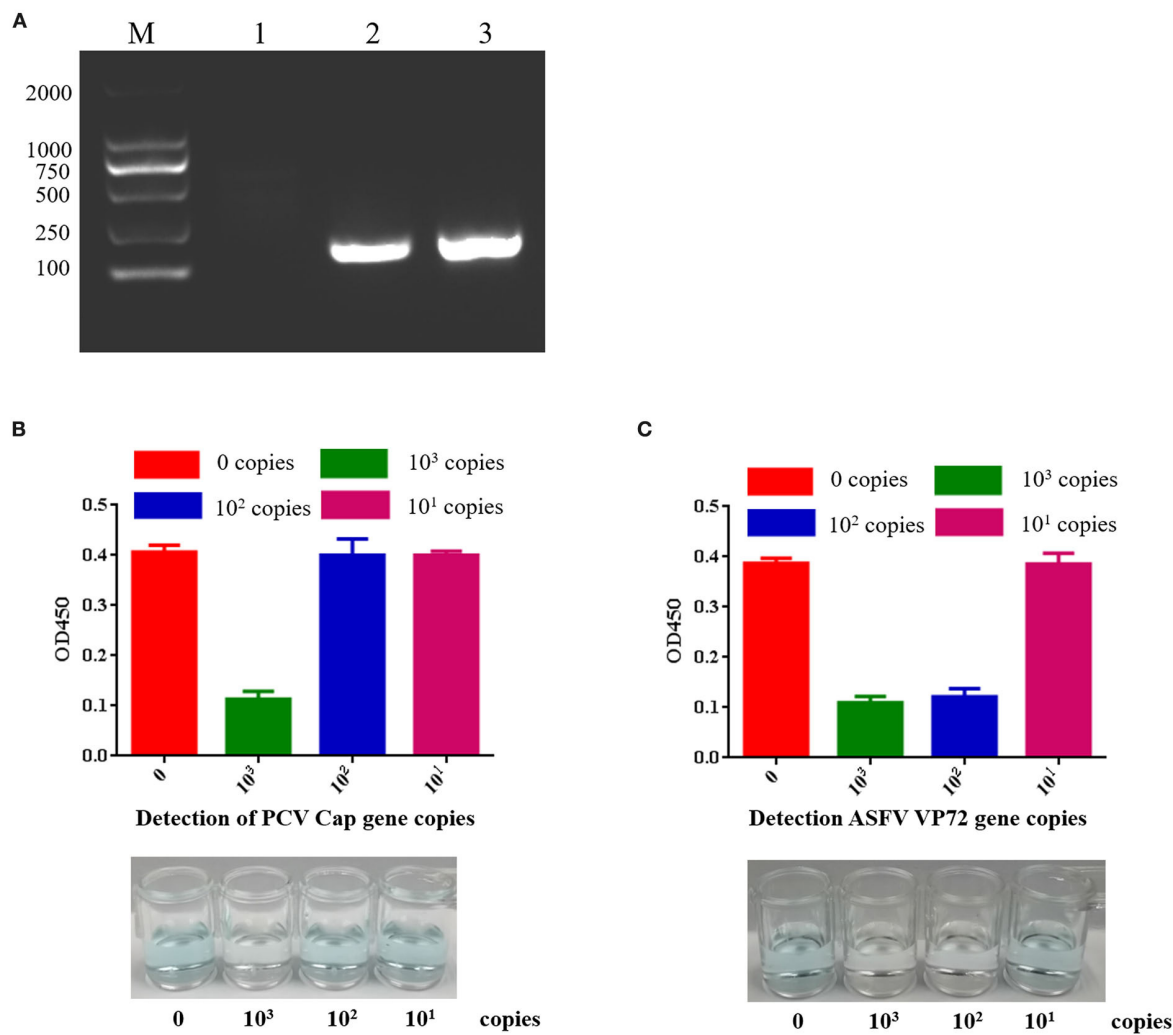
Sensitivity of PCV2 and ASFV DNA detection by CRISPR-Cas12a combined with G-quadruplex. **(A)** RPA amplified target DNA. Lane M: 2000 DNA ladder; Lane 1: negative control; Lane 2: RPA amplification of PCV2 target sequence; Lane 3: RPA amplified ASFV target sequence. **(B)** PCV2 DNA detection by CRISPR-Cas12a combined with G-quadruplex. The least copies of this method were 10^3^ copies. **(C)** ASFV DNA detection by CRISPR-Cas12a combined with G-quadruplex. The least copies of this method were 10^2^ copies.

Cas12a nucleic acid detection scheme is shown in Figure 3. RPA was used to amplify clinical samples *in vitro* (25). TwistAmp^®^ Basic was applied for amplification. PCV2 Cap gene and ASFV VP72 gene were amplified by RPA (Figure 4A). PCV2 Cap and ASFV VP72 DNA template were adjustment to 1.0 × 10^3^, 1.0 × 10^2^, and 1.0 × 10^1^ copies, and 2 μL of DNA template was added for RPA amplification. The RPA maximum amplification is 1.0 × 10^3^ copies for the PCV2 Cap gene (Supplementary Figure S6), and 1.0 × 10^2^ copies for the ASFV VP72 gene (Supplementary Figure S7).

MagBead DNA Purification Kit is used for purifying DNA fragments, 20 μL of ddH_2_O resuspend and recover DNA fragments. The 18 μL RPA reaction product was added with 2.5 μL of reaction buffer (10×), 1 μL of crRNA at a final concentration of 40 ng/μL, 2.5 μL of 1 μM LbCas12a (Cpf1) at a final concentration of 100nM, and 1 μL of G-quadruplex and incubated in 37 °C 45 min. 2.0 μM hemin and 100 μL TMB were added and incubated for 20 min. Green-colored oxidized product was used for DNA detection (Figures 4B,C). If the reaction fluid is green, it indicates no target DNA, while colorless indicates target DNA. The results showed that the least copies of PCV2 DNA detected by CRISPR-Cas12a combined with G-quadruplex was 10^3^ copies, and the least copies number of ASFV DNA detected by CRISPR-Cas12a combined with G-quadruplex was 10^2^ copies. Our results indicated that G-quadruplex could be used as a substrate for color reaction, nucleic acid could be detected without an instrument, and the reaction could be carried out at room temperature. This method can be used for field diagnosis with high detection speed and accuracy in poor conditions.

## Discussion

The African swine fever virus, a double-stranded DNA virus with a genome size of 170–193 kb, belongs to the ASF family (26). The virus has five unique structural features, including an outer membrane, capsid, double inner membrane, core–shell, and genome. The virus particle contains more than 30,000 protein subunits and is assembled into spherical particles with a diameter of about 260 nm. Most of the virus surface is composed of the main capsid protein VP72 (27). At present, there are no effective vaccines for the prevention of ASF, so a rapid detection for effective diagnoses of ASFV is conducive to the purification of ASF.

At present, there are no specific treatments for PCV2. It is easy for it to be coinfected with other viruses, such as classical swine fever virus (CSFV), porcine reproductive and respiratory syndrome virus (PRRSV), and porcine pseudorabies virus (PRV), thus brings great difficulties to the diagnosis (28). The virus was detected according to clinical symptoms, and mixed infection was easy to lead to missed detection of the PCV2 virus. Some methods have been developed for PCV2 detection. An isothermal RPA assay has been established (29). In addition, the droplet digital polymerase chain reaction (30) and TB green II-based duplex real-time fluorescence quantitative PCR assay (31) were applied to detect PCV2 and PCV3.

African swine fever continues to mutate in China, and there is no effective vaccine yet. Therefore, the farms need to find it early and report it to the authorities as soon as possible, to kill and disinfect the pigs in the epidemic area, and to better limit the spread of the virus. As an immunosuppressive disease, PCV2 needs to be isolated and eliminated from infected pigs. This method can promote the diagnosis of infected pigs and assist the management of pig farms.

At present, the detection of ASFV and PCV2 has been reported in many methods and with high sensitivity. The detection sensitivity of DNA extraction-free qPCR, visual LAMP, and fluorescent LAMP assays for the detection of ASFV could detect 5.8 copies/μL, the same as qPCR (32). The RPA-Cas12a-fluorescence assay can be detected with as few as two copies of ASFV (33). The One-pot platform for rapid detecting viruses utilizing RAA and CRISPR/Cas12a could detect 3.07 copies/μL (34). The detection sensitivity of EvaGreen real-time PCR combined with melting curve analysis could detect 5.0 copies/μL (35). LAMP-coupled CRISPR-Cas12a can be detected with a low detectable limit of 1 copy/μL of PCV2 (36). In this study, the detection sensitivity of ASFV and PCV2 was 10^2^ copies and 10^3^ copies, respectively, which are lower than the above detection methods. The above methods, especially the qPCR method, which is the gold method for virus detection, require instruments and are not conducive to field detection. Our method can well avoid this problem. It does not rely on instruments but only on reaction in tubes. Our method can directly observe the results with naked eyes, which is not available in the above methods.

The HlyA gene is by hemin/G-quadruplex DNAzyme and hybridization chain reaction signal amplification (37). G-quadruplex-based biosensors have a particular focus on SARS-CoV-2 detection (38). The detection of patulin toxin is by using DNA G-quadruplex with aggregation-induced emission (39).

African swine fever and porcine circovirus are two infectious diseases that China's breeding industry pays more attention. The rapid detection of ASF is helpful for the farms to report the epidemic situation to the authorities. After the official diagnosis, the pigs in the farms will be harmlessly treated as soon as possible to limit the spread of ASFV. Porcine circovirus infection leads to the decline of pig immunity, and it is easy to infect other bacterial or viral infectious diseases. The method we studied can quickly detect ASF and circovirus type 2 without relevant instruments. At present, this method is aimed at DNA viruses and mainly relies on the DNA amplification of RPA. It cannot detect RNA vaccines. Therefore, it is necessary to develop further the RNA virus detection method based on this study.

## Conclusion

At present, ASF is spreading in China and European countries, and farms need to find and report quickly to better limit the spread of the virus. PCV2 is an immunosuppressive disease. This method can speed up the diagnosis of infected pigs and isolate and eliminate infected pigs. In this study, effective detection of ASFV and PCV2 nucleic acid as the target gene is achieved. In addition, this method can complete nucleic acid detection at 37 °C without using other instruments or energy. Our research has expanded the application of Cas12a and laid the foundation for the field rapid detection of viral nucleic acid in future.

## Data availability statement

The original contributions presented in the study are included in the article/Supplementary material, further inquiries can be directed to the corresponding author.

## Author contributions

YW contributed to the investigation, visualization, and writing of original draft. YW, RL, and YZ contributed to the methodology. YW, WZ, and SH contributed to the project administration. ZL contributed to the resources, supervision, validation, and writing of review's editing. All authors contributed to the article and approved the submitted version.

## Funding

This work was funded by the Key Science and Technology Project of Guangxi (Gui Ke AA18118051 to ZL) and the National Key Research and Development Program of China (2018YFD0500800 to ZL).

## Conflict of interest

The authors declare that the research was conducted in the absence of any commercial or financial relationships that could be construed as a potential conflict of interest.

## Publisher's note

All claims expressed in this article are solely those of the authors and do not necessarily represent those of their affiliated organizations, or those of the publisher, the editors and the reviewers. Any product that may be evaluated in this article, or claim that may be made by its manufacturer, is not guaranteed or endorsed by the publisher.

## Supplementary material

Supplementary Figure S1 Determination of the optimum hemin concentration. Red curve with 1mM G4; Green curve without G4.

Supplementary Figure S2 Determination of the optimum G-quadruplex concentration. Red curve with 2 μM hemin; Green curve without hemin.

Supplementary Figure S3 Target gene amplification. Lane M: 2000 DNA marker; Lane 1: negative control; Lane 2: PCV2 Cap protein gene PCR amplification; Lane 3: ASFV VP72 protein gene PCR amplification.

Supplementary Figure S4 RPA primers and crRNA design of PCV2 Cap gene.

Supplementary Figure S5 RPA primers and crRNA design of ASFV VP72 gene.

Supplementary Figure S6 Sensitivity of RPA for PCV2. Different copies of PCV2 DNA were used as template for RPA. The PCV2 lowest copies that could be determined by RPA was 10^3^ copies. Lane M: 2000 DNA marker; Lane 1: Negative control; Lane 2: 10^3^ copies; Lane 3: 10^2^ copies; Lane 4: 10^1^ copies.

Supplementary Figure S7 Sensitivity of RPA for ASFV. Different copies of ASFV DNA were used as template for RPA. The ASFV lowest copies that could be determined by RPA was 10^2^ copies. Lane M: 2000 DNA marker; Lane 1: Negative control; Lane 2: 10^3^ copies; Lane 3: 10^2^ copies; Lane 4: 10^1^ copies.

Supplementary Table S1 Sequence information for primers.
