# Supplementary figures and images for: Visual and label-free ASFV and PCV2 detection by CRISPR-Cas12a combined with G-quadruplex
Source: Front Vet Sci. 2022 Nov 29;9:1036744. doi: 10.3389/fvets.2022.1036744 (PMC9745048; doi:10.3389/fvets.2022.1036744)

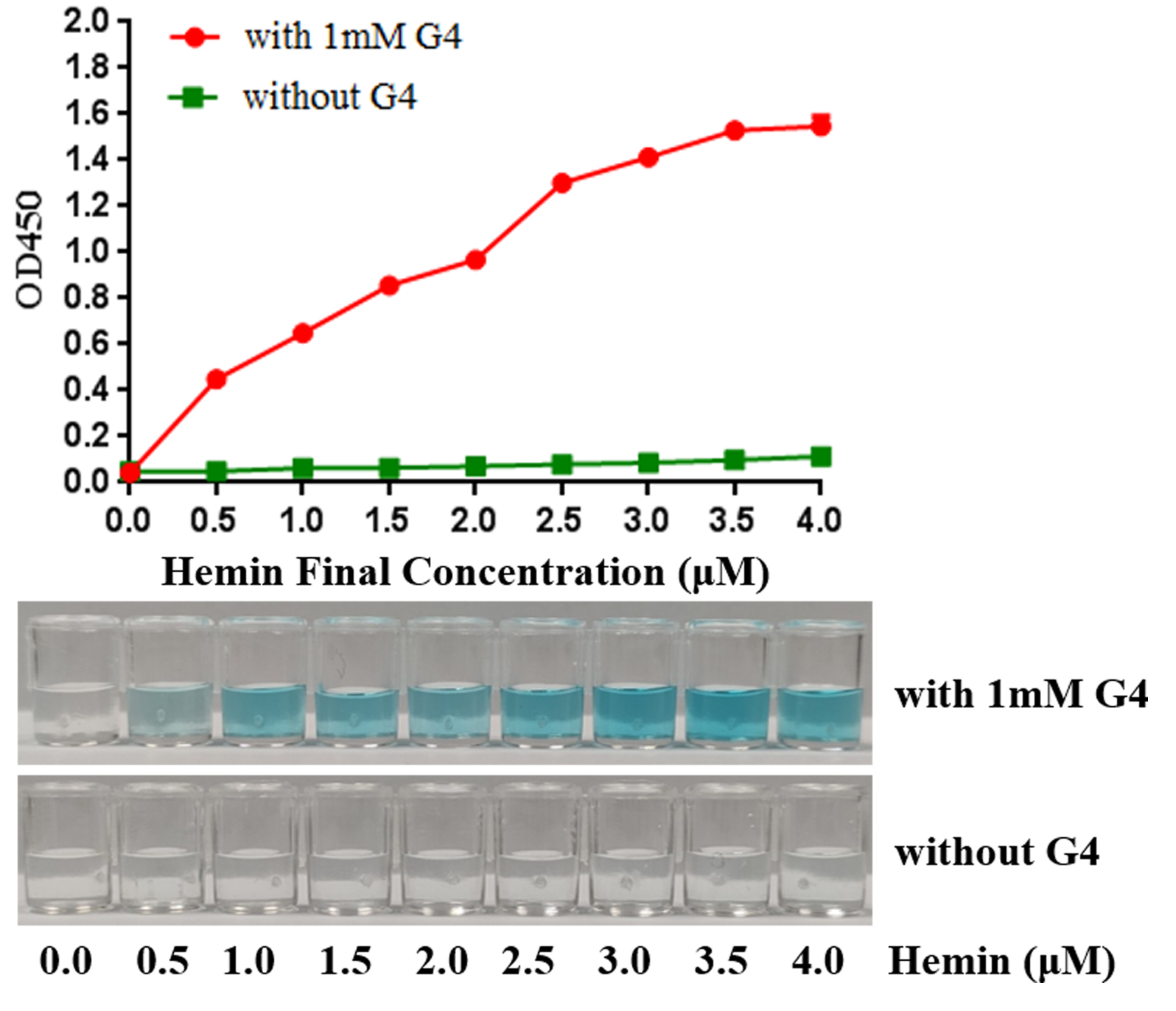

Supplement: Supplementary Figure S1 — Determination of the optimum hemin concentration. Red curve with 1mM G4; Green curve without G4. [file Image_1.TIF]

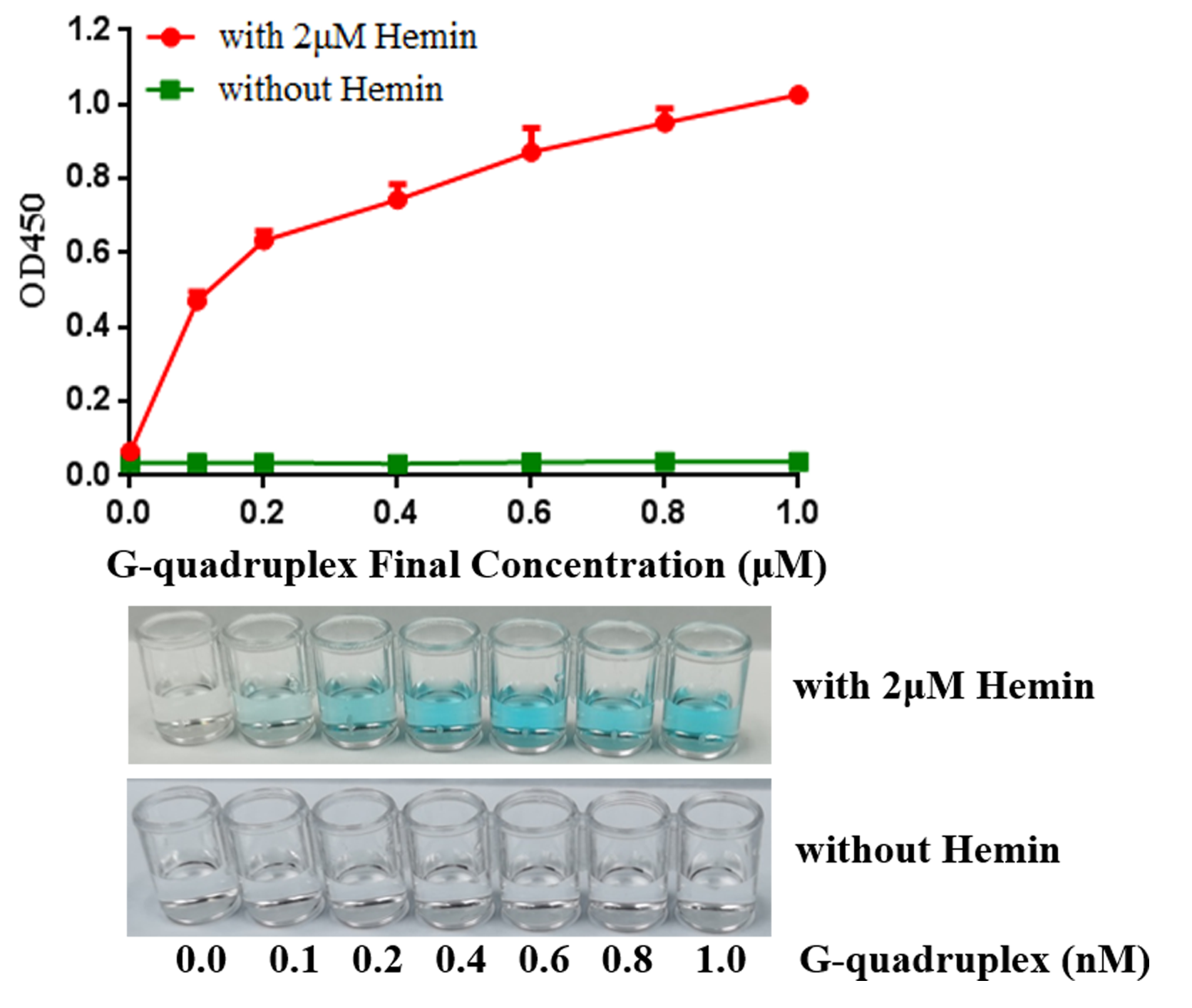

Supplement: Supplementary Figure S2 — Determination of the optimum G-quadruplex concentration. Red curve with 2 μM hemin; Green curve without hemin. [file Image_2.TIF]

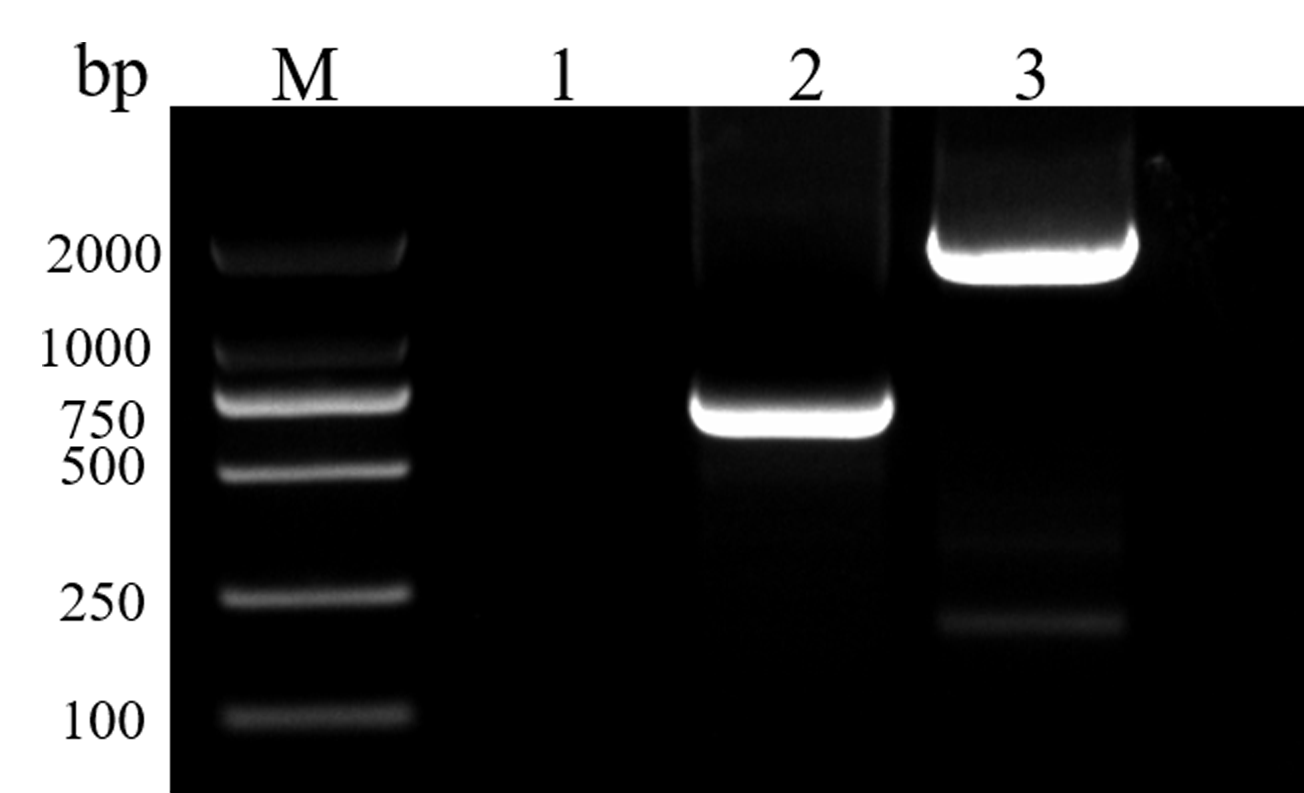

Supplement: Supplementary Figure S3 — Target gene amplification. Lane M: 2000 DNA marker; Lane 1: negative control; Lane 2: PCV2 Cap protein gene PCR amplification; Lane 3: ASFV VP72 protein gene PCR amplification. [file Image_3.TIF]

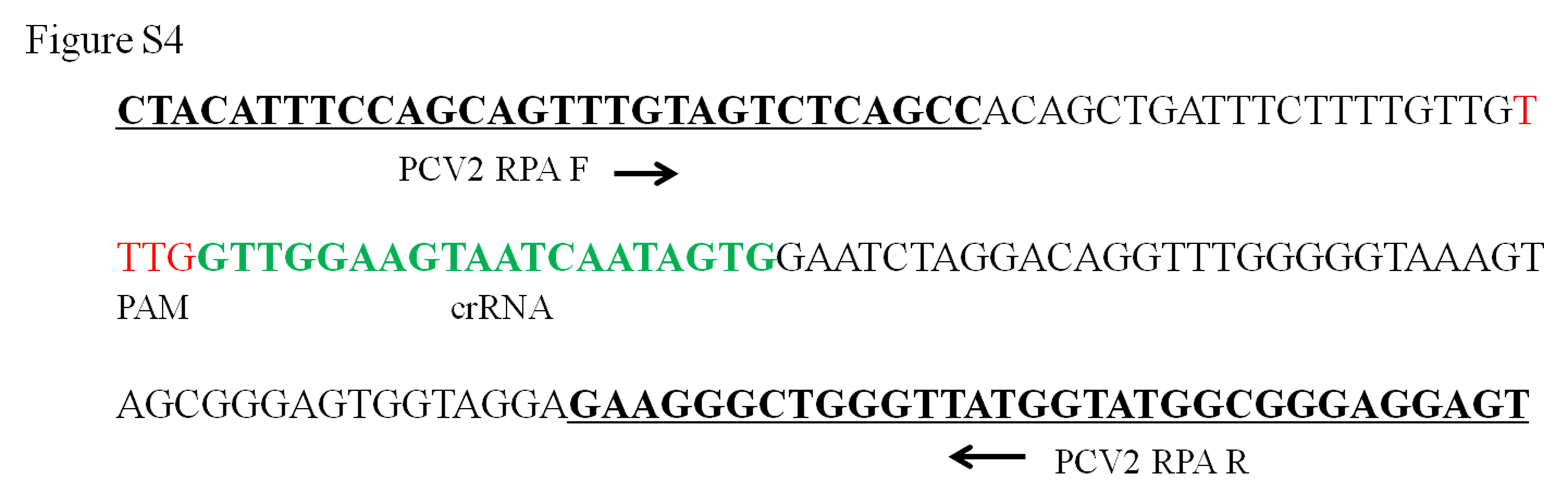

Supplement: Supplementary Figure S4 — RPA primers and crRNA design of PCV2 Cap gene. [file Image_4.TIF]

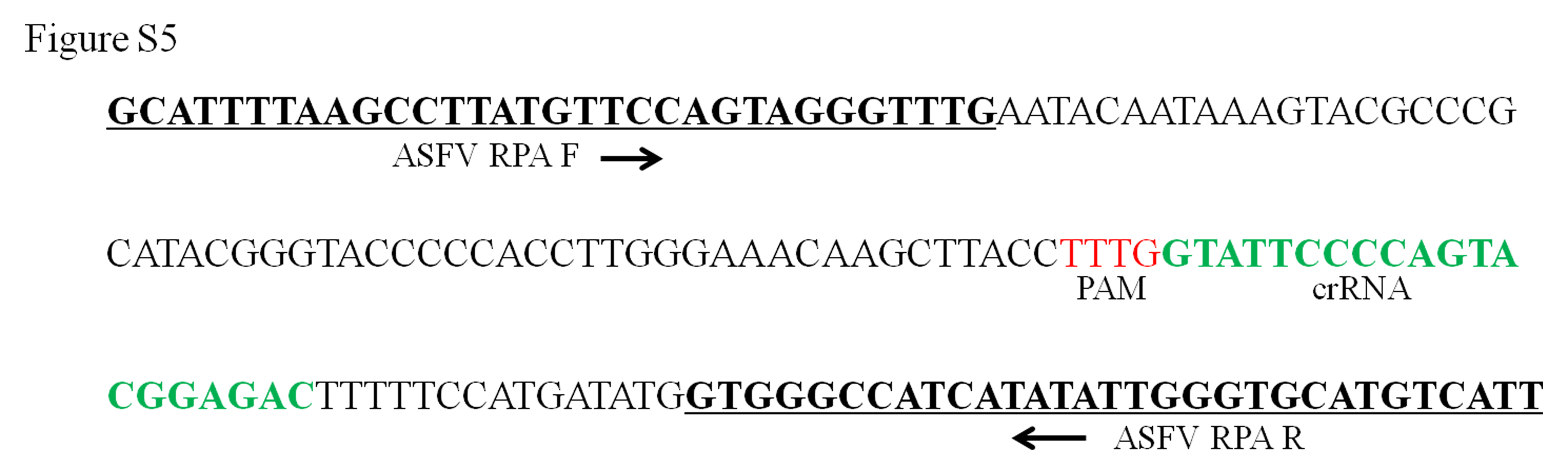

Supplement: Supplementary Figure S5 — RPA primers and crRNA design of ASFV VP72 gene. [file Image_5.TIF]

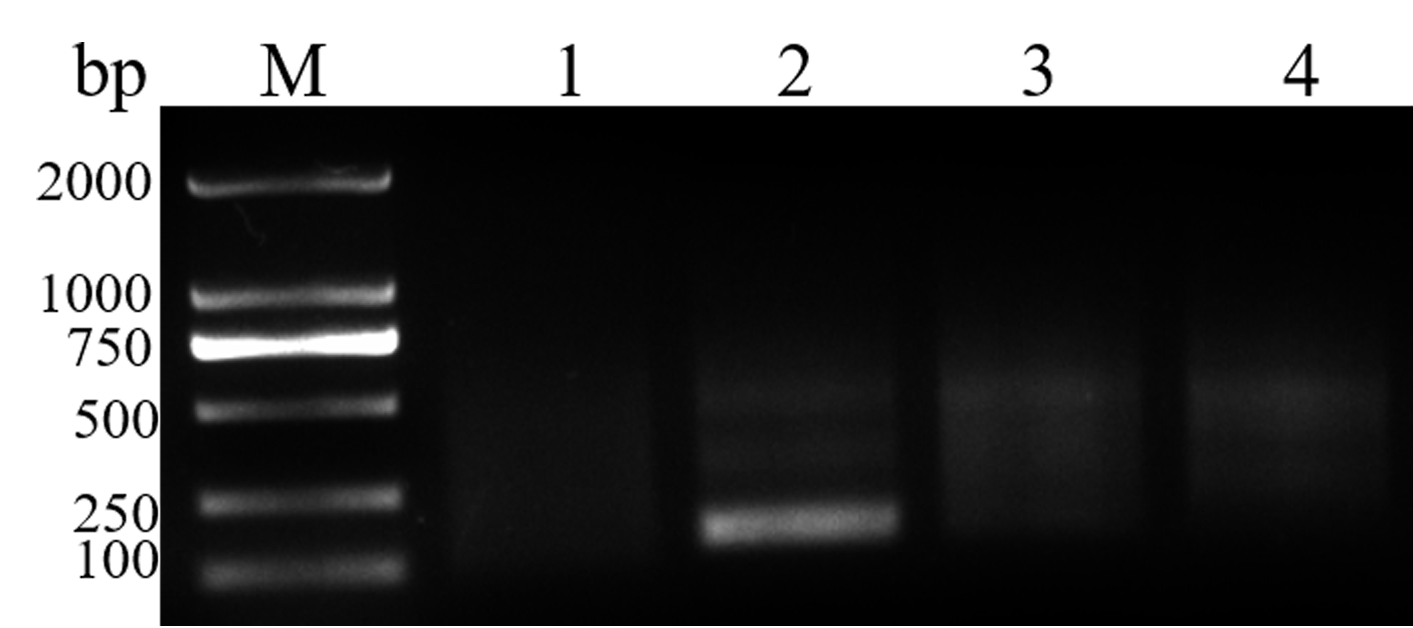

Supplement: Supplementary Figure S6 — Sensitivity of RPA for PCV2. Different copies of PCV2 DNA were used as template for RPA. The PCV2 lowest copies that could be determined by RPA was 103 copies. Lane M: 2000 DNA marker; Lane 1: Negative control; Lane 2: 103 copies; Lane 3: 102 copies; Lane 4: 101 copies. [file Image_6.TIF]

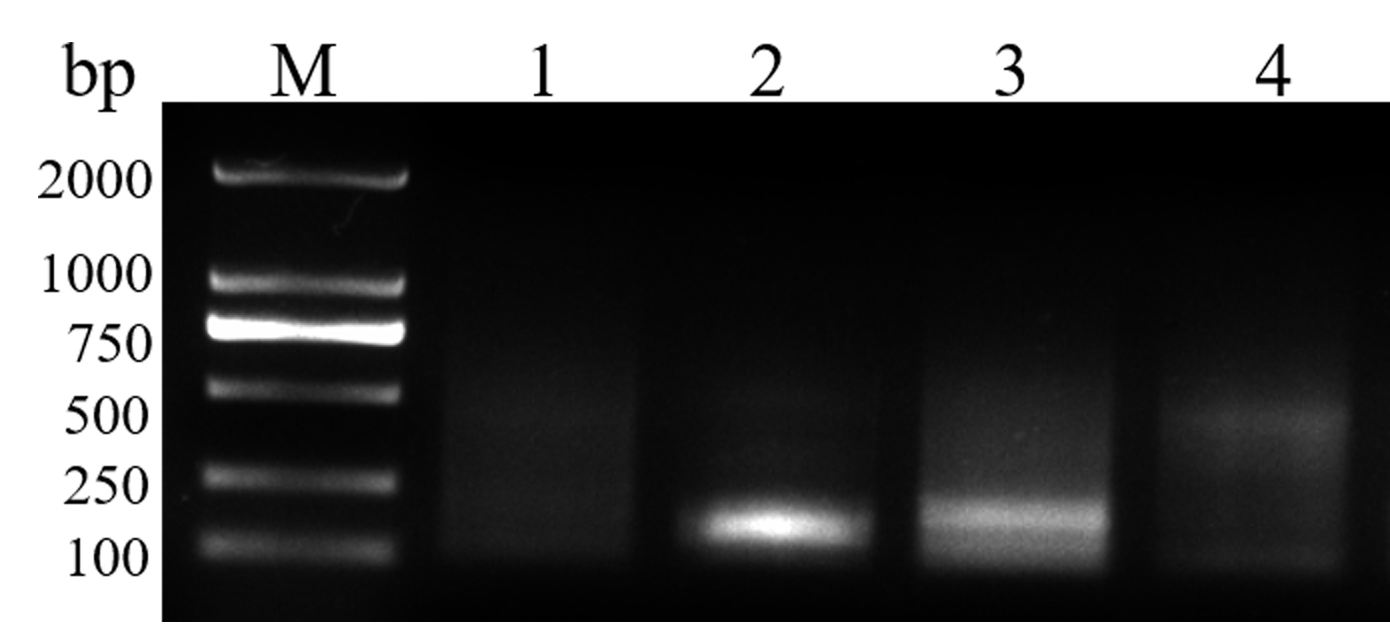

Supplement: Supplementary Figure S7 — Sensitivity of RPA for ASFV. Different copies of ASFV DNA were used as template for RPA. The ASFV lowest copies that could be determined by RPA was 102 copies. Lane M: 2000 DNA marker; Lane 1: Negative control; Lane 2: 103 copies; Lane 3: 102 copies; Lane 4: 101 copies. [file Image_7.TIF]
