# Supplementary material for: Visual and label-free ASFV and PCV2 detection by CRISPR-Cas12a combined with G-quadruplex
Source: Front Vet Sci. 2022 Nov 29;9:1036744. doi: 10.3389/fvets.2022.1036744 (PMC9745048; doi:10.3389/fvets.2022.1036744)
Supplement: Supplementary Table S1 — Sequence information for primers. [file Data_Sheet_1.PDF]

Table S1. Sequence information for primers.

Name	Sequence(5'-3')
PW17	GGGTAGGGCGGGTTGGG
PCV2 Cap F	ATGACGTATCCAAGGAGGCGTTACCGGAGAAGAAG
PCV2 Cap R	TTAAGGGTTAAGTGGGGGGTCTTTAAGATTAAATTC
ASFV VP72 F	TTAGGTACTGTAACGCAGCACAGCTGAACCGTTC
ASFV VP72 R	ATGGCATCAGGAGGAGCTTTTTGTCTTATTGCTAAC
crRNA F	GAAATTAATACGACTCACTATAGGGTGGATAATTTCTACTGTTGTAGAT
PCV2 crRNA R	CACTATTGATTACTTCCAACATCTACAACAGTAGAAATTATCCA
ASFV crRNA R	GTCTCCGTACTGGGGAATACATCTACAACAGTAGAAATTATCCA
PCV2 RPA F	CTACATTTCCAGCAGTTTGTAGTCTCAGCC
PCV2 RPA R	ACTCCTCCCGCCATACCATAACCCAGCCCTTC
ASFV RPA F	GCATTTTAAGCCTTATGTTCCAGTAGGGTTTG
ASFV RPA R	AATGACATGCACCCAATATATGATGGCCCAC
